# When microbial biotechnology meets material engineering

**DOI:** 10.1111/1751-7915.13975

**Published:** 2021-11-24

**Authors:** Ana M. Hernández‐Arriaga, Cristina Campano, Virginia Rivero‐Buceta, M. Auxiliadora Prieto

**Affiliations:** ^1^ Polymer Biotechnology Group, Department of Plant and Microbial Biotechnology Biological Research Centre Margarita Salas Spanish National Research Council (CIB‐CSIC) Madrid Spain; ^2^ Interdisciplinary Platform for Sustainable Plastics towards a Circular Economy‐CSIC (SusPlast‐CSIC) Madrid Spain

## Abstract

Bacterial biopolymers such as bacterial cellulose (BC), alginate or polyhydroxyalkanotes (PHAs) have aroused the interest of researchers in many fields, for instance biomedicine and packaging, due to their being biodegradable, biocompatible and renewable. Their properties can easily be tuned by means of microbial biotechnology strategies combined with materials science. This provides them with highly diverse properties, conferring them non‐native features. Herein we highlight the enormous structural diversity of these macromolecules, how are they produced, as well as their wide range of potential applications in our daily lives. The emergence of new technologies, such as synthetic biology, enables the creation of next‐generation‐advanced materials presenting smart functional properties, for example the ability to sense and respond to stimuli as well as the capacity for self‐repair. All this has given rise to the recent emergence of biohybrid materials, in which a synthetic component is brought to life with living organisms. Two different subfields have recently garnered particular attention: hybrid living materials (HLMs), such as encapsulation or bioprinting, and engineered living materials (ELMs), in which the material is created bottom‐up with the use of microbial biotechnology tools. Early studies showed the strong potential of alginate and PHAs as HLMs, whilst BC constituted the most currently promising material for the creation of ELMs.

## Graphical abstract

When microbial biotechnology meets material engineering: bringing bacterial biopolymers to life.

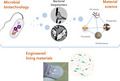

## Introduction

Biopolymers are macromolecules produced naturally by living organisms, such as plants, animals and microorganisms with very diverse chemical and physical structures. In the last decade, bacterial biopolymers have drawn much attention due to their suitability for production in sustainable bioprocesses and to the fact that their properties can be tailored with the use of microbial biotechnology tools. This has promoted the development of a new generation of bio‐based materials with applications in key fields such as biomedicine (i.e. drug delivery systems, tissue‐repairing materials, tissue scaffolds, etc.) (Moradali and Rehm, 2020; Blanco *et al*., 2021). Likewise, their unique properties such as biodegradability, renewability and biocompatibility substantiate their enormous potential for use in other applications like food packaging, biosensors, biometrics or bioplastics. In terms of the circular economy, bacterial biopolymers are extremely important in the present context of diminishing petroleum‐based materials and the global environmental damage caused by the use of synthetic materials.

In view of all this, researchers have established different strategies for developing novel biomaterials based mainly upon engineering bacteria to produce biopolymers and on the diversification of the biomaterial through chemical modification of its side chains or by blending or cross‐linking with other biopolymers/molecules. Over the last few years, there has been extensive development of metabolic engineering approaches that make use of microbial cells as factories. In addition, the recent advances in omic techniques provide an understanding of biosynthesis and degradation pathways, regulatory and signalling systems and of the complex circuits of enzymes and regulators involved in the metabolism of bacterial biopolymers (Mezzina *et al*., 2021). In this sense, metabolic engineering, synthetic biology and computational modelling have allowed the design of bacterial processes that increase production yields and/or generate polysaccharides, polyamides, polyphosphates and polyhydroxyalkanoates (PHAs) possessing functionalities. Current trends highlight the potential of bacterial biopolymers for use as biomaterials that host living organisms, with the ultimate objective of creating engineered living materials (ELMs), in which the material can self‐repair, and sense and respond to stimuli (Nguyen *et al*., 2018).

In the present paper, we first review the structural diversity, properties and applications of bacterial biopolymers, as well as the current trends in material science attempting to diversify its applications by means of chemical and physical approaches. Herein, we will discuss how the new materials can be ‘brought to life’ through the incorporation of living organisms. The latter would confer smart properties to the material that are typical of living systems like plants. To achieve this ambitious goal, microbial biotechnology needs to work in synch with material engineering. Hence, the present article will discuss, in a critical context, the potentialities of the most promising bacterial biopolymers, whilst considering the requirements of both scientific fields.

## The bacterial biopolymers: ‘who’ they are and how they are produced

Bacteria produce natural polymers as part of their inherent physiology in the form of storage molecules, protective capsular layers surrounding cells, as a major component of biofilm or as an extracellular matrix that externally protects the bacterial population from the environment (Rehm, 2010). The main advantage of bacterial biopolymers in comparison to polymers extracted from other natural sources is that their physicochemical properties can be tailored for a specific application with the use of biotechnology tools. With this bottom‐up approach, the molecular weight, charge, monomer composition and specific 3D structure can all be modified, resulting in different thermo‐chemical and mechanical properties. Intensive research efforts have, therefore, focused upon improving production yields, as well as the physicochemical properties thereof (Blanco *et al*., 2021).

**Fig. 1:**
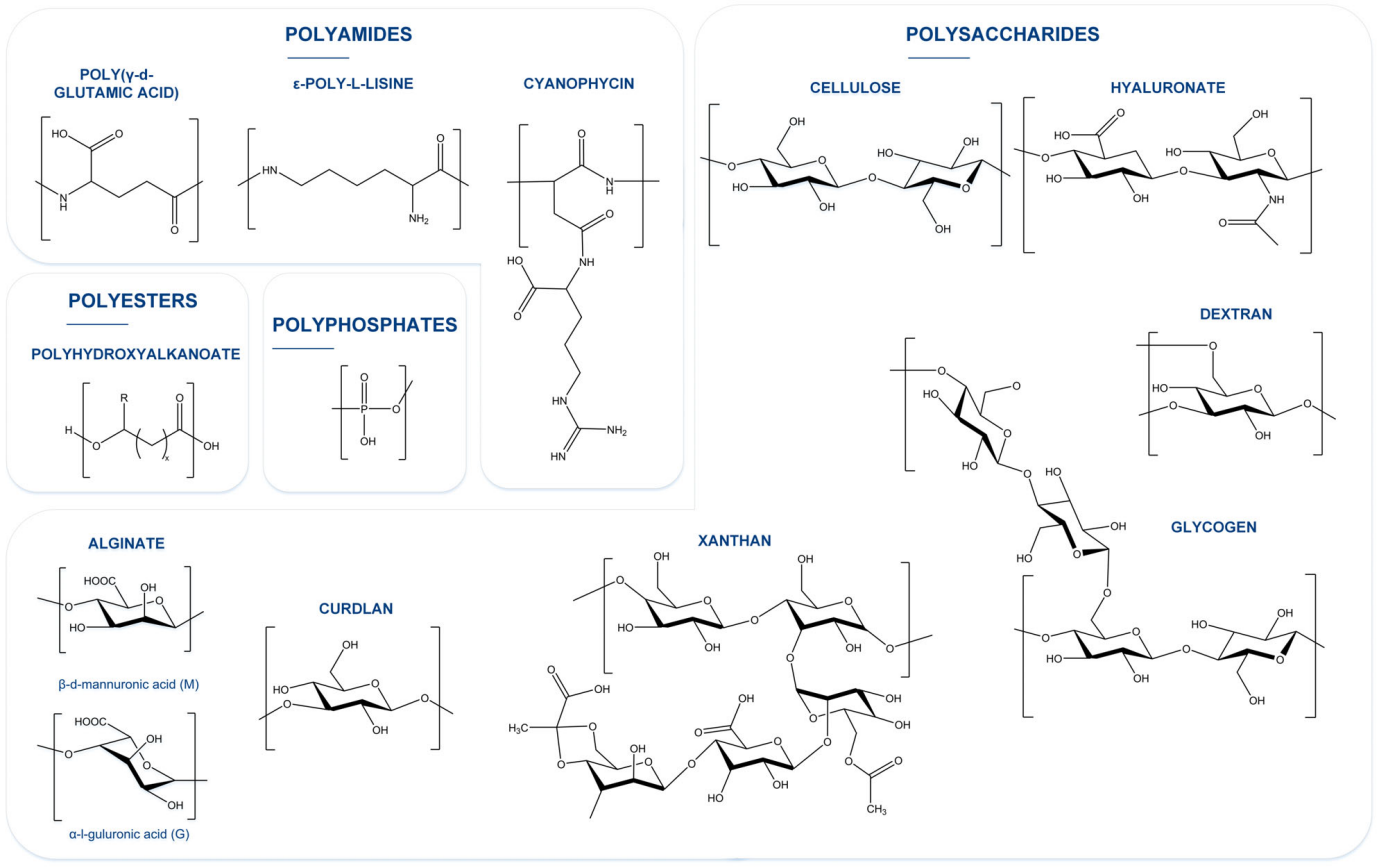
Chemical structure of the most abundant and applied bacterial biopolymers.

Bacteria can synthesize various types of biopolymers with different chemical compositions, 3D configurations and yields. Apart from natural proteins and nucleic acids, they can produce polyamides (Oppermann‐Sanio and Steinbüchel, 2002), polyesters (Mezzina *et al*., 2021), polyphosphates (polyP) (Rosigkeit *et al*., 2021), polysaccharides (Schmid *et al*., 2015) and extracellular recombinant proteins with applications in the polymer biotechnology field (Smith *et al*., 2017). Figure 1 shows the chemical structure of the most abundant and commonly used bacterial biopolymers, whilst Table 1 summarizes the species of bacterial producers, the location of the polymers in relation to the cells during production and their major applications.

**Table 1:** Key bacterial biopolymers and their applications as bio‐based materials.

Polymer/cell location	Main producer strains	Major field of applications
γ‐PGA/Ext	(N) *Bacillus* spp. (R) *B. subtilis*; *B. licheniformis* strains, and *E. coli*	Biomedical applications as drug carriers; food industry and bioremediation as dispersing agent and food additives
ε‐PL/Ext	(N) *Streptomyces* spp. (R) *Streptomyces albulus* sp.	Biomedical applications as antimicrobial coatings, fibres and drug carriers; industrial applications, as food preservatives
Cph/Int	(N) *Cyanobacteria* spp. (R) *Cyanobaceteria spp*, *E. coli*, *Corynebacterium glutamicum, R. eutropha*	Industrial applications as biologically active compounds, biofertizantes and bioplastics; source of amoniacids for pharmaceutical industry
PolyP/Int	(N) Most bacteria (R) *Citrobacter freundii*	Biomedical applications, regenerative medicine and drug delivery; industrial applications for delivering phosphate for synthesis reactions
PHAs/Int	(N) Scl‐PHA *C. necator*, *Halomonas* spp.; Mcl‐PHA *Pseudomonas* spp. (R) *P. putida*, *C. necator* and *E. coli*	Biomedical, regenerative medicine, implants and tissue engineering applications as nanoparticles, fibres, films, blends and composites. Bioplastics
BC/Ext	(N) *E. coli*, *P. fluorescens*, *K. hansenii, K. xylinus, K. medellinensis*; and *K. rhaeticus* iGEM (R) *E. coli*	Biomedical applications as drug delivery, cell encapsulation, wound dressing, tissue engineering; industrial applications such as acoustic and food, textile industry
Dextran/Ext	(N) *Lactobacillus brevis*, *Leucononostoc mesenteroides,* (R) *L. mesenteroides*	Therapeutic drug delivery systems, tissue engineering scaffolds and food and cosmetic additives
Curdlan/Ext	(N) Bacteria from the genera *Rhizobium*, *Agrobacterium*, *Alcaligenes*, and *Bacillus* (R) *Agrobacterium* sp	Tissue engineering scaffolds, drug encapsulation, and food additive
Alginate/Ext	(N) *Pseudomonas* ssp. and *Azetobacter* spp. (R) *E. coli*	Biomedical applications for drug delivery, cell encapsulation and tissue engineering; environmental remediation and food additive
HA/Ext	(N) *Streptococcus* spp.*, Pasteurella multocida* and *B. cereus* G9241 (R) *S. zooepidemicus*, *L. lacti*s, *B. subtilis* and *E. coli* and C*. glutamicum*	Hydrogels, nanoparticles and microparticles for many medical, pharmaceutical, food and cosmetic applications
Xanthan/Ext	(N) *Xanthomonas* spp. (R) *X. campestris*	Therapeutic carrier, drug delivery systems, food additives
Glycogen/Int	(N) Bacteria and Archaea (R) *Synechocistic* sp. PCC6803	Biomedical applications as solubilizing agent and nanocarrier for therapeutic drugs
Bacterial curli amyloide fibres/Ext	(N) *E. coli*, *Salmonella*, *Citrobacter* and *Enterobacter* spp. (R) *E. coli*	Scaffold for material engineering; reinforce composites for biomedical applications
Bacterial collagen‐like proteins/Ext	(N) *S. pyogenes*; Extremotolerant bacteria (R) *E. coli*	Scaffold for tissue engineering, wound healing, cosmetics
Silk protein/Ext	(R) *E. coli*, *Cyanobacteria*	Artificial fibre spinning; composites for wound healing and skin regeneration; antibacterial coatings for biomedical applications

N, natural producers; R, recombinant host producers.

Cell location: Ext, indicate extracellular; Int, indicate intracellular.

### Polyamides

Polyamides comprise a group of amino acid polymers in which monomers are linked by peptide bonds. They can be synthetized via ribosome‐dependent pathways, as natural or recombinant proteins such as curli fibres, collagen, fibronectin and silk (Fig. 1 and Table 1), but also can be synthesized in a non‐ribosome‐dependent pathway known as polyaminoacids or polyamides (Fig. 1 and Table 1). To date, only three different polyaminoacids have been described in bacteria: poly‐γ‐glutamic acid (PGA) and ε‐poly‐l‐lysine (ε‐PL), synthesized inside the cells and secreted into the culture medium, and cyanophycins (Cph), accumulated as intracellular granules. Polyamides can be highly charged and may be polyanionic (PGA), polycationic (ε‐PL) or neutral (Cph) (see reviews about those families below).

PGA is a linear extracellular polyamide consisting of L, D, or both enantiomers of glutamic acid. It connects the α‐amino and γ‐carboxyl groups by means of amide bonds. It is biodegradable, water‐soluble, edible, and non‐toxic, and could, therefore, present interesting applications in industry such as thickeners, bitterness‐relieving agents, cryoprotectants, drug carriers, biopolymer flocculants and heavy metal absorbers amongst others (Nair *et al*., 2021). It is produced by diverse bacteria of the *Bacillus* species, and its physiological function is believed to depend on the microorganism environment. Some of the bacillary species can synthesize the PGA attached to the peptidoglycan layer (i.e. *Bacillus anthracis*), in which case PGA is considered to represent an important virulence factor. The principal species exploited for the industrial production of PGA are able to release the biopolymer into the medium. This group includes *Bacillus subtilis*, *Bacillus licheniformis*, and *Bacillus amyloliquefaciens*. The proposed microbial biosynthetic pathway of PGA in *B. subtilis* involves l‐glutamic acid units supplied exogenously or endogenously by means of α‐ketoglutaric from the TCA cycle as a direct precursor. The synthesis of PGA is catalysed by an enzyme complex, the γ‐PGA synthetase, encoded by the *pgs* operon (Cao *et al*., 2018). Some researchers have focused upon optimizing growth conditions in order to increase yield, manipulate enantiomeric composition and to engineer the molecular mass of the polymer. Different heterologous hosts have been postulated as effective PGA producers, including *Escherichia coli* and *Corynebacterium glutamicum*.

Another polyamide of interest is ε‐PL due to its broad antibacterial spectrum, water solubility, edibility, non‐toxicity and good thermal stability. It has been used as a food preservative, an emulsifying agent, a dietary agent, drug carriers, an anticancer agent enhancer, biochip coatings, or an adhesive subbing (Chen *et al*., 2021). Furthermore, ε‐PL is a linear homopolyamino acid, with a polycationic character, typically comprising 25–35 identical l‐lysine (lys) residues with amide linkages between the ε‐amino and α‐carboxyl groups. ε‐PL is naturally produced and released into the medium by many soil microorganisms, where the Gram‐positive bacterium *Streptomyces albulus* ssp. lysinopolymerus strain 346 produces the highest yield (Wang *et al*., 2020). The industrial production of ε‐PL is currently conducted by aerobic fermentation involving a mutant derived from *S. albulus* 11011A. Several attempts have been made to improve ε‐PL production yields, for example with the use of additives or by replacing the carbon sources affecting polymer chain length. Recently, the production of genetically and metabolically engineered strains has enabled a greater yield of ε‐PL by cloning or mutating the key genes such as ε‐PL synthetase gene (*pls*) (Chen *et al*., 2021).

Finally, Cph can be defined as polyamides comprising poly l‐aspartic acid backbone and L‐arginine side groups linked via isopeptide bonds with a branched structure (Fig. 1). Due to the interest in the abovementioned monomers, Cph have mainly been produced as an intermediate product to be further cleaved. Cph are produced by most cyanobacteria species including unicellular and filamentous ones and by some heterotrophic bacteria, under stress conditions (stationary phase light or temperature) (Kumar *et al*., 2015).

Cph accumulate in the form of granules inside the cells as a nitrogen storage reservoir and are largely insoluble under physiological conditions. They are synthesized in a non‐ribosomal‐dependent pathway from aspartic acid and arginine precursors by the cyanophycin synthetase (CphA1) and are degraded to aspartate and arginine by the coordinated enzymatic activity of cyanophycinase (CphB) and isoaspartyl dipeptidase (Du *et al*., 2019). The metabolism of Cph has been widely reported, and although there is limited productivity of the Cph obtained by native producers at the industrial‐scale, different biotechnological strategies have been employed to improve the process; examples of these involve optimization of the production media with additives (arginine) or renewable carbon sources, downstream processes, as well as the heterologous expression in various microbial strains of diverse cyanobacterial Cph synthetases (CphAs). Apart from cyanobacteria, Cph are produced by some heterotrophic bacteria used for industrial production, such as *Acinetobacter* sp. DSM 587, *E. coli*, *C. glutamicum*, *Cupriavidus necator* and *Pseudomonas putida* (Watzer and Forchhammer, 2018).

### Polyphosphates

PolyP is an inorganic linear polymer of phosphate residues (Pi), linked by energy‐rich phosphoanhydride bonds. This biopolymer is highly anionic and is widely used as a reagent in water treatment, fertilizers, flame retardants and food additives in view of its unique properties, inexpensiveness, non‐toxicity and biodegradability (Sanz‐Luque *et al*., 2020). It has been reported to be present in all life forms from bacteria to mammals. Bacteria accumulate PolyP granules under growth in unbalanced media and/or stressful environmental conditions (Rosigkeit *et al*., 2021). PolyP possesses a whole range of functions in organisms ranging from energy storage to different stress responses. Analysis of polyP‐deficient mutants has also demonstrated its relevance in virulence, motility, biofilm formation, and cell cycle control: Additionally, the PolyP metabolism has been studied in several model organisms such as *E. coli*, *P. aeruginosa*, *C. necator* and *Cyanobacteria* spp. (Sanz‐Luque *et al*., 2020; Rosigkeit *et al*., 2021). It is synthesized in bacterial cells by PolyP kinases (PPK1 and PPK2) and degraded by exopolyphosphatase (PPX). Biotechnological production of Polyps has been described for the genera *Mycobacterium* and *Corinebacterium*; Moreover, cyanobacteria seen to possess enzymes involved in the cyanobacterial polyP metabolism, and mechanisms for metabolism regulation have been established (Sanz‐Luque *et al*., 2020).

### Polyesters

**Fig. 2:**
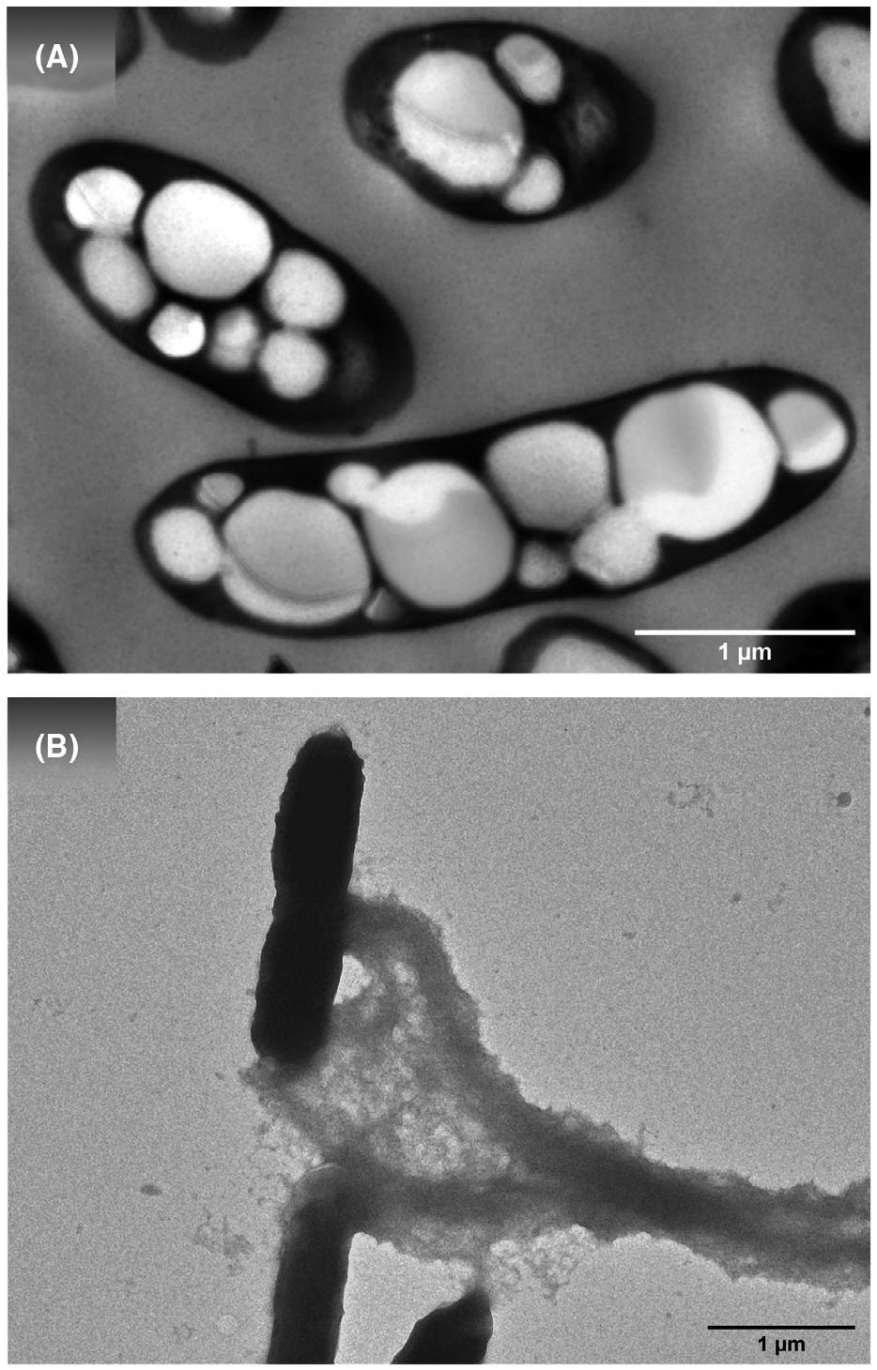
Examples of an intracellular and extracellular biopolymer. A. PHA accumulated in *Pseudomonas putida* KT2440 cells and B. BC nanofibrils extruded from *K. medellinensis* cells.

Polyhydroxyalkanoates (PHAs) consist of thermoplastic linear polyesters of several (*R*)‐3‐hydroxyacid ((R)‐HA) monomer units connected by an ester bond. Although the most habitual substituent groups found in PHA monomers are aliphatic chains, great variability can be achieved by controlling the metabolism involved in PHA accumulation. The material properties of PHAs, such as hydrophobicity, melting point, glass transition temperature and degree of crystallinity, are highly dependent on monomer composition, the length of the PHA side chain, and on its chemical nature (Choi *et al*., 2020). To date, approximately 150 different monomer constituents have been described, including unsaturated aliphatic and aromatic groups, amongst others, in the lateral chain, a fact that indicates the great potentiality of these biopolymers. PHAs are usually classified according to their monomer size as short‐chain‐length PHA (scl‐PHA), consisting of 3–5 carbon monomers such as poly(3‐hydroxybutyrate) or PHB, a homopolymer made up of C‐4 monomers; medium‐chain‐length PHA (mcl‐PHA), which contains 6–14 carbon monomers; and long‐chain length (lcl‐PHA), comprising 15 carbon monomers or longer. No natural PHAs containing monomers hydroxylated in four or two positions have been so successfully produced by microbial biotechnology strategies (Choi *et al*., 2020). In general terms, PHAs are hydrophobic, and present good resistance to hydrolytic attack and to UV; they sink in water, which facilitates anaerobic biodegradation in sediments. In addition, they are biocompatible and some of them are biodegradable, whilst others are compostable. Hence, PHAs are postulated as a green alternative to petroleum‐based plastics (Tan *et al*., 2021) in applications ranging from adhesives to medical implants (Choi *et al*., 2020; Mezzina *et al*., 2021). These polymers are stored inside the cells of gram‐positive and gram‐negative bacteria under both aerobic and anaerobic conditions and have been described as carbon and energy stores impacting the whole bacterial physiology (Prieto *et al*., 2016) (Fig. 2A).

*Cupriavidus necator* H16 (scl‐PHA) and *P. putida* KT2440 (mcl‐PHA), are model strains employed for the study of the PHA metabolism as well as to enhance biotechnological production. Biosynthesis and accumulation of PHAs constitutes a complex and global metabolic process involving the central carbon metabolism of the bacteria where β‐oxidation is a key step for PHA biosynthesis from structurally related precursors (Mezzina *et al*., 2021). The biosynthesis cycle of PHAs depends on the activity of enzymes such as PHA synthases (PhaC) and depolymerases (PhaZ), which degrade PHAs, releasing (*R*)‐HAs that can be used as carbon and energy sources. Differences between PHA structures are mainly due to the enzymatic specificity of the PHA synthase, the key enzyme involved in PHA biosynthesis, which can accept precursors of a certain carbon‐length, as well as the substrate employed, along with the metabolic and regulatory networks in each species. The specific aspects of the metabolic and regulatory pathways involved in the PHA biosynthesis/depolymerization cycle have been described and reviewed (Mezzina *et al*., 2021; Mitra *et al*., 2021).

### Polysaccharides

Bacterial polysaccharides encompass an enormous group of structurally diverse molecules with a linear or branched structure that includes a wide range of the same carbohydrate monomers or of different types, linked by glycosidic bonds. According to their composition, they are classified as homopolymers and heteropolymers and can be neutral or charged (most of them are polyanionic). Some examples of homopolymer bacterial polysaccharides refer to bacterial cellulose (BC) and dextran, whilst alginate, hyaluronic acid or xanthan are majorly heteropolymers (Moradali and Rehm, 2020; Rana and Upadhyay, 2020). Different bacteria produce polysaccharides, storing them inside the cells (for example, glycogen) or secreting them either as capsular polysaccharides linked to the cell surface (for example, the K30 antigen) or as free exopolysaccharides that contribute to the formation of the biofilm matrix (i.e. alginate, cellulose, hyaluronic acid, xanthan, dextran).

Exopolysaccharides or extracellular polysaccharides are produced by most species of bacteria as an extracellular matrix component (ECM) of biofilms, known as potentially structured communities of bacterial cells (Flemming *et al*., 2016). The interaction of polysaccharides and other polymeric substances such as proteins and extracellular DNA can determine the properties of the biofilm matrix and, therefore, the survival of the cells in diverse environments. Specific functions of exopolysaccharides depend upon structural composition and host environment (Rana and Upadhyay, 2020).

Amongst bacterial biopolymers, BC has aroused particular interest due to the highly porous fibre network formed during the static culture of bacteria, with a water holding capacity of up to 400 times its dry weight. The BC fibres comprise from hundreds to thousands of linear chains of β (1 → 4) linked d‐glucose units (Jacek *et al*., 2019). The high crystallinity of BC, together with its high purity, renewability, biodegradability and biocompatibility, make this material unique for certain applications with high quality requirements, such as those in the biomedical or pharmaceutical industry as well as for biotechnological purposes (Campano *et al*., 2016; Portela *et al*., 2019).

BC is produced as an extracellular polymer by some genera of bacteria, like *Agrobacterium*, *Pseudomonas*, *Rhizobium* and *Sarcina*, whilst strains belonging to the *Komagataeibacter* genus are noteworthy for their high productivity (see below) (Fig. 2B). The biochemical and genetic mechanisms, as well as the regulation pathways of BC biosynthesis have all been characterized in the model strain *K. xylinus* (Ryngajłło *et al*., 2020). BC is synthetized in a multi‐step process by an enzymatic complex that includes four proteins, that is BcsA, BcsB, BcsC and BcsD, which are encoded by the bacterial operon *bsc* (Jacek *et al*., 2019). The polymerization proceeds with the phosphorylation of glucose to glucose 6P and isomerization to glucose 1P, which incorporates a uridine phosphate group (UDP) to convert glucose 1P to UDP‐glucose, the substrate for Bcs synthase (Jacek *et al*., 2019). The efficiency of BC production and the properties of BC depend upon growth medium composition, growth cultivation conditions and on the BC producer strain. Several strategies have been developed to improve BC yields for industrial production such as selection of strains with a high capacity for BC production, optimization of growth conditions and bioengineering approaches. Although *K. xylinus* is the model organism, the highest yield of BC from this strain is obtained from culture media supplemented with glucose, which is expensive at the industrial‐scale. BC yield can be improved by including additives in the growth medium such as glycerol, agar, xanthan, sodium alginate, ethanol or carboxymethyl cellulose (CMC).

Other relevant bacterial exopolysaccharides include alginate and hyaluronate. Alginate is an anionic biopolymer comprising mannuronic and guluronic acid blocks with a high water retention capacity, which makes it form a dense hydrogel matrix. Its properties, such as solubility, hydrophobicity, and biological functionality, depend on the monomeric composition sequence, molecular weight and on the attached functional groups. These properties can be altered by modifying the availability of hydroxyl, as well as the carboxyl groups in its structure (Szabó, *et al*., 2020a). Bacterial alginates are produced as an exopolysaccharide in several *Azotobacter* spp. and *Pseudomonas* spp. To date, however, the commercial production of alginates has been obtained from brown algae. Alginate hydrogels are of great interest in pharmaceutical and biomedical applications because of their porosity, swelling, biodegradability, biocompatibility and non‐antigenicity. Furthermore, alginate has been approved by the U.S. Food and Drug Administration (FDA) and is widely used as a biomaterial in pharmaceutics, regenerative medicine and dentistry.

Finally, hyaluronate is a biocompatible and biodegradable mucoadhesive polysaccharide, also approved by the U.S. FDA; it is present in the extracellular matrix and articulations of mammals. It is produced by *Streptococcus pyrogens* and *Bacillus cereus* to protect against phagocytosis. Hyaluronic acid presents a negative charge and is used in polyelectrolyte complex formations with other oppositely charged polymers. It is also used as a copolymer for drug delivery, in wound healing, tissue regeneration, ophthalmic treatment, intra‐articular injections, etc. Modified hyaluronic acid has been reported for its application as a dental implant, ocular lenses, catheters, dermal regeneration, etc (Moradali and Rehm, 2020; Szabó, *et al*., 2020a).

## Diversifying biopolymer applications

As described above, bacterial biopolymers are highly diverse both in composition and in 3D structure and they exhibit a wide range of possibilities for the production of biotechnologically tailored biomaterials with applications in different scientific fields and industries. According to the particular production process, the properties of each biopolymer depend on the sequence of monomer composition, molecular weight and on its macromolecular assembly. However, diversification of biopolymers by means of introduction of hydrophilic, acidic, basic or other functional groups, as well as through the interaction between the reactive groups and other biomolecules, can improve the properties of these bio‐based materials, making them suitable for added‐value applications. These biomaterials can be diversified by means of biotechnology and synthetic biology, bioengineering, bio‐computing and with chemical modification approaches. These techniques can be combined to achieve cutting‐edge materials with enhanced properties for specific applications, which facilitates the creation of a wide range of biomaterials. In particular, with chemical modification methods, such as blending, grafting/cross‐linking and curing, the properties of those biopolymers can be tailored (López Durán *et al*., 2018).

Blending is a simple and low‐cost strategy for tailoring polymer properties; materials are formed through physical interactions on mixing different polymers. This procedure is usually performed via solvent‐casting and melt‐compounding methods and has been widely used to enhance properties for specific purposes. This strategy is highly relevant in the particular case of PHAs, as it facilitates their biomedical application (Lukasiewicz *et al*., 2018; Rivero‐Buceta *et al*., 2020). Indeed, the biocompatibility of films made of P(3HB)/P(3HB‐co‐3HHx) inoculated with fibroblast was observed to be enhanced in relation to those solely made up of P(3HB) due to the fact that crystallinity was reduced (Kai *et al*., 2003). In another example, a slow and controlled release of tetracycline (TC) was demonstrated with the use of a drug delivery carrier made up of copolymers of PHA. Herein, P(3HB‐HV) microspheres blended with low‐molecular‐weight polyvinyl alcohol as a surface stabilizer resulted in the highest TC loading, providing a slow release for effective periodontal treatment against *Actinobacillus actinomycetemcomitans* and *Porphyromonas gingivalis* (Panith *et al*., 2016). This method was also used to obtain anti‐staphylococcal hydrogels based on BC and the antimicrobial PHA biopolyester known as poly(3‐hydroxy‐acetylthioalkanoate‐co‐3‐hydroxyalkanoate) (PHACOS). The novel PHACOS20 (BC 80%–PHACOS 20%) hydrogel show mechanical and thermal properties within the range of human skin; it also exhibited good anti‐staphylococcal activity (kills 1.8 logs), proving to constitute a good candidate for wound‐healing applications (Rivero‐Buceta *et al*., 2020).

Blending can also be performed with other natural biopolymers; for instance, the properties of the positively charged chitosan (CS) can be enhanced significantly following the addition of polyanionic γ‐PGA, making it more hydrophilic and cytocompatible for tissue engineering applications (Hsieh *et al*., 2005).

Grafting is another chemical modification consisting of covalently binding molecules or other polymers to a polymer chain; it produces modified (or newly conferred) physicochemical or functional properties in the resulting material. Different methods have been developed to obtain graft copolymers, such as chemical, photochemical, radiation, plasma‐induced and enzymatic techniques. For example, the abundance of functional groups on the surface of some bacterial biopolymers, for example polysaccharides makes them susceptible to modification, transforming them into carboxylic acid, amine, aldehyde or thiol groups. Further modification of these groups could lead to the grafting of a wide range of molecules, such as proteins, polymers, metal nanoparticles, and antibiotics (Tavakolian *et al*., 2020).

One of the most commonly researched approaches attempts to enhance the antimicrobial features of the biopolymers to be used in biomedical applications. For example, BC can be modified with aminoalkyl groups via a silane chemical grafting approach, which resulted in aminaded cellulose. This modification conferred antimicrobial properties against *Staphylococcus aureus* and *E. coli* and was non‐toxic to human adipose‐derived mesenchymal stem cells, whilst mechanical and thermal properties were further enhanced (Fernandes *et al*., 2013). Another research team grafted antibiotics such as amoxicillin onto a pH‐responsive polyacrylic acid‐BC composite (Chuah *et al*., 2018). Antimicrobial CS has also been chemically grafted onto BC by oxidation with NaIO_4_ to dialdehyde BC and Schiff’s base reaction with the amino group of CS, achieving a maximum functionalization ratio of 12.38% (wt) (Liu *et al*., 2019). In the case of PHA‐based materials, grafting of small molecules such as vinyl imidazole onto poly‐3‐hydroxyoctanoate (P(3HO)) resulted in antibacterial activity against *E. coli* and *S. aureus* (Chung *et al*., 2012); furthermore, P(3HB) grafted with 1,2 ethylenediamine provided the same results.

Some authors have treated the surface of some biopolymers with plasma, which is a grafting technique consisting of dispersion of an ionized gas; the procedure modifies the functional groups on the surfaces due to the high reactivity of the ions and electrons. Interestingly, it only modifies the surface layer without affecting the bulk properties of the polymer. In addition, P(3HB) treated with Ar plasma augmented surface polarity by increasing the number of oxygen containing groups, which resulted in improved cell adhesion, proliferation and spreading homogeneity on the PHB surface (Slepička *et al*., 2018).

Adhesiveness of PHA was enhanced by the introduction of a fibronectin active fragment (GRGDS peptide) by a multi‐step synthesis (Tajima *et al*., 2016). Additionally, Na‐alginate was functionalized by grafting with vinyl sulfonic acid in the presence of a thiourea/peroxydiphosphate system or with polyacrylamide under microwave irradiation to improve its effectiveness as a flocculant for water treatment applications (Szabó *et al*., 2020a).

Moreover, oligo peptides, glycyl‐l‐glutamine or glycyl‐glycyl‐glycine, were grafted by another research team onto BC, thus enhancing its interfacial wettability and boosting the mineralization induction, as well as improving the affinity between polymeric and mineral phases. BC was oxidized with sodium periodate (NaIO_4_) to produce dialdehyde BC, and oligo peptides were grafted via Schiff’s base reaction with their amino group, followed by reduction of the imine group through addition of NaBH_4_ (Sun *et al*., 2019).

In the case of cross‐linking, monomers are covalently bonded onto a polymer chain in the presence of cross‐linkers, forming tridimensional networks. Cross‐linkers are the key drivers of this technique; for instance, antimicrobial BC‐CS blends were formed by incubation of BC with a CS solution for 24 h and further stabilized by means of tripolyphosphate cross‐linking. Further antimicrobial activity was achieved by ciprofloxacin impregnation, also resulting in a biocompatible material, as revealed by in vitro studies in human fibroblasts (Cacicedo *et al*., 2020). Also, the antimicrobial character of BC was improved with ε‐PL, which was covalently conjugated to CMC adsorbed to the BC by means of carbodiimide chemistry. In this approach, greater capacity for water retention was observed, whilst the viscoelastic properties showed no changes (Fürsatz *et al*., 2018).

This technique was also used to produce PHA‐based scaffolds for medical applications. The unsaturated copolyester poly[(*R*)‐3‐hydroxybutyrate‐co‐(*R*)‐3‐hydroxy‐10‐undecenoate] (PHBU) was cross‐linked via thiol‐ene click chemistry. The resulting PHA scaffold showed enhanced tensile strength, insignificant cytotoxicity and biocompatibility in human mesenchymal stem cells; it presented properties closer to those relevant for soft tissue replacement (Levine *et al*., 2016).

In another example, Yang *et al* developed a type of polypeptide‐based hydrogel comprising functionalized PGA through Michael‐addition reactions. Firstly, two PGA‐derivatives were prepared PGA‐conjugated cysteamine (PGA‐SH) and methacrylate‐PGA (PGA‐GMA) (Yang *et al*., 2020). Subsequently, the hydrogel was accomplished by means of the Michael‐addition reaction between thiol groups (PGA‐SH) and methacrylate groups (of PGA‐GMA) in a mild cross‐linking process. The physicochemical properties of the hydrogel were tailored by means of adjustment of the copolymer concentrations. The PGA hydrogel exhibited superior biocompatibility and high stability. Moreover, following the *in vitro* and *in vivo* experiments, the artificial PGA hydrogels were able to mimic the native ECM, which can provide friendlier circumstances for stem cells *in vivo*.

In addition, multilayer structures based on thermoplastic corn starch nanocomposites/BC nanowhiskers (BCNW) films and PHA/BCNW coatings were developed. The methodology used, by combining nanocomposites and multilayer design, resulted in good adhesion between the layers and enhanced barrier performance (Fabra *et al*., 2016).

Finally, some polymers were modified by means of the curing technique, in which an oligomer polymerizes to form a coating that adheres to the substrate by physical forces. The most popular curing method for polymer composites involves thermal curing, which is widely used for preparing biocomposites in industry, including the automotive, construction and furniture sectors. These biopolymers are reinforced with other compounds such as zirconia (Zhang *et al*., 2018) in order to provide better mechanical properties. Another example was a novel material based on PHA, which exhibits a good shape‐memory effect and rapid recovery: PHA was combined with segments of polyurethane (PHP), telechelic‐hydroxylated polyhydroxyalkanoate (PHA‐diols) and polyethylene glycol (PEG), which confer thermo‐responsive and water‐responsive properties. The curing process enabled the material to be shape‐morphing, self‐folding and to take on three‐dimensional (3D) scaffolds, making this material a potential candidate for biomedical applications (Wang *et al*., 2019). Light curing was used to create a nanogel for controlled drug release from methacrylated PGA (Bakó *et al*., 2016).

## Towards engineered living materials: bringing bacterial biopolymers to life

Next‐generation cutting‐edge materials should present smart functional properties, ideally those of living systems present in nature, such as adaptation to environmental cues, an ability for self‐healing; they should be able to sense and respond to stimuli and to dynamically switch between different material states (Nguyen *et al*., 2018). Integration of genetically programmed living cells into non‐living scaffolds is currently enabling the reliable use of a wide‐ranging repertoire of functionalities of living cells (at macroscopic scale and outside of their natural environments) for multiple technological applications (Liu *et al*., 2017). These materials, fabricated bottom‐up, have been classified as biohybrid materials, a term that refers to any composite material that has a biological and a synthetic component (Smith *et al*., 2020). Biological components include purified molecules, such as proteins or DNA, or living cells, whilst synthetic components can either involve organic or inorganic polymers, minerals, ceramics or even metals.

A subfield of biohybrid materials was recently introduced as hybrid living materials (HLMs) (Smith *et al*., 2020). HLMs are intended to interface genetic engineering with material manufacturing platforms in order to solve the shortcomings of controllability currently existing in biohybrid materials. Some examples include the direct printing of cells with hydrogels (i.e. bioprinting) (Rutz *et al*., 2015), seeding cells in specific positions of tailor‐made scaffolds (de Koster *et al*., 2015), and engineering cells to endogenously grow the structural materials these inhabit (Mukherjee *et al*., 2018). The most recent emerging area within biohybrid materials are the ELMs. ELMs have been defined as engineered materials consisting of biological systems that assemble the material, as they are autonomously patterned, and which modulate the functional performance of the material in some manner (Gilbert and Ellis, 2019). The advantage of ELMs over other biohybrid materials, in which the synthetic component is produced prior to functionalization with bacteria is that, apart from sensing and responding to their environment, ELMs can self‐repair (Gilbert and Ellis, 2019).

### Hybrid living materials based on bacterial biopolymers

Researchers have focused their attention on engineering new biomaterials using different biotechnological and chemical strategies enabling the incorporation of bioactive molecules, such as cells, fragments of cells, proteins, enzymes or even DNA, for the production of HLMs (Bilal and Iqbal, 2019). To this end, bacterial‐derived biopolymers such as alginate, hyaluronic acid, cellulose, PHAs and protein‐based polymers including collagen, fibronectin and silk have been investigated. Some of them have been engineered prior to contact with the biological component in order to enhance some of their features, such as mechanical and physical properties, biocompatibility, water retention capacity and resistance to chemicals; or they have also been modified through addition of organic or inorganic components to obtain additional functionalities in the resultant HLMs. Since cell immobilization requires the physical containment of cells in a polymeric matrix without loss of desired biological activity, the properties of polymeric matrices are crucial in these applications. Different strategies can be envisaged depending on the type of cell, the biochemical and biophysical properties of the immobilizing polymeric matrix, and the type and field of the application to be addressed, such as adsorption of cells by materials, their adhesion to a surface, entrapment in 3D polymeric matrix or encapsulation of cells.

Cell encapsulation is a bioengineering technology, mainly applied in biomedicine; it usually requires a biocompatible matrix covered by a semi‐permeable membrane, which protects the inner cells from the host immune response and mechanical stresses. The efficacy on the encapsulation and the viability of the encapsulated microorganisms also depends on the properties of the biomaterials and the encapsulation method (Razavi *et al*., 2021). Biopolymers such as alginate, hyaluronic acid or fibrin collagen have been approved for encapsulating cells for application in therapeutic delivery systems, alginate being the most used for biomedical applications (Coelho‐Rocha *et al*., 2018). For example, microencapsulation of therapeutic cells for the mitigation of inflammatory response following transplants has been achieved via modification of sodium alginate (Na‐alginate) hydrogels with PEG derivatives, without restriction of their gelling abilities. The microspheres maintained the favourable properties of alginate gels but presented enhanced performance in terms of *in vivo* durability and physical properties (Szabó *et al*., 2020b). A highly promising approach involves electrospinning, a technique that uses polymer solutions and strong electric fields to produce nano‐sized fibres that offer wide‐ranging applications such as carriers of microorganisms, stem cells, proteins, and nucleic acids in therapeutic and other applications (Stojanov and Berlec, 2020). Encapsulating bacteria in alginate‐based electrospun nanofibres for probiotics has also been widely applied (Diep and Schiffman, 2021).

Apart from alginate, several biodegradable polymeric matrices have been tested for bacteria encapsulation, such as CMC, poly‐lactic (PLA) and poly‐lactic‐co‐glycolic acid (PLGA), CS, and mixtures of proteins and polysaccharides. Bacterial polyesters, PHAs, have recently emerged as biodegradable polymers for developing microcarrier devices. PHAs have been widely studied in the field of tissue engineering and in the development of medical devices and they are also of great interest in the production of controlled release formulations. In recent times, PHAs have been used to immobilize antimicrobial biosystems based on bacteriophages (Wang *et al*., 2016) and bacterial predators such as *Bdellovibrio bacteriovorus* (González *et al*., 2020). With potential bioremediation and industrial applications, the PHA‐microencapsulation method has also been extended to other key microorganisms in microbial biotechnology, such as *P. putida* KT2440 (González *et al*., 2020).

Despite these remarkable features, the programmable biochemical machinery of bacteria for the creation of HLMs with controlled three‐dimensional (3D) shape, microstructure, and dynamic metabolic response is an extremely attractive cutting‐edge technology in which much remains to be explored, designed and implemented.

### Material science, microbial biotechnology and synthetic biology as drivers for engineered living materials

The widespread development of synthetic biology in combination with computational system biology and gene sequence platforms (Tang *et al*., 2021) have provided advanced techniques, new methods and sophisticated tools for genetically engineering living cells and other biological systems in order to produce novel and useful materials with new and programmable properties. Indeed, synthetic biology has become one of the most important drivers for the design and development of ELMs (Heyde *et al*., 2017; Nguyen *et al*., 2018; Liu and Xu, 2020).

As explained above, ELMs can be defined as biological materials that self‐assemble via a bottom‐up process (Gilbert *et al*., 2021). In this sense, study of ELMs focuses, on the one hand, on the use of engineered organisms that are reprogrammed to simultaneously create the material, conferring to it novel functionalities to serve as ‘in situ’ growing functional biomaterials. On the other hand, the living cell component of the ELMs can also be genetically engineered and redesigned to sense and respond to stimuli, resulting in living materials presenting emerging and programmable functionalities (Liu and Xu, 2020; Gilbert *et al*., 2021; Rivera‐Tarazona *et al*., 2021). Suitable integration of these living components with non‐living components constitutes an important and challenging task with regard to maintaining the long‐term stability of the ELMs and, therefore, their new functionalities. Consequently, the combination of synthetic biology in the context of microbial biotechnology with the tools of material science has promoted the development of living systems; these involve dynamic and responsive materials with programmable capabilities such as living sensors, living therapeutics and electronics, as well as energy‐conversion materials and also living building materials (Heveran *et al*., 2020; Gilbert *et al*., 2021; Shang *et al*., 2021).

Synthetic biology enables the living cells to be engineered, whether it be those forming the biological component of ELMs or the ones that produce the synthetic component. Microorganisms such as *E. coli*, *K. rhaeticcus*, *B. subtilis*, *P. putida*, *Cyanobacteria* model strains, and yeasts such as *S. cerevisiae* have been successfully engineered to provide new and programmable properties by means of a wide range of novel synthetic biology tools for creating synthetic microbial chassis. For example, *E. coli* bacterial biofilms have been engineered with the use of a molecular programming strategy consisting of several peptide domains genetically fused to the amyloid protein CsgA, one of the main components in *E. coli* biofilms. These engineered CsgA fusion proteins are successfully secreted and extracellularly self‐assembled into amyloid nanofibre networks or curli fibres that retain the functions of the tagged peptide domains displayed. Based on this strategy, *E. coli* biofilms have been engineered into a chemical‐inducer‐responsive electrical switch (Chen *et al*., 2014) or in a biofilm‐integrated nanofibre display system (Nguyen, 2017).

**Fig. 3:**
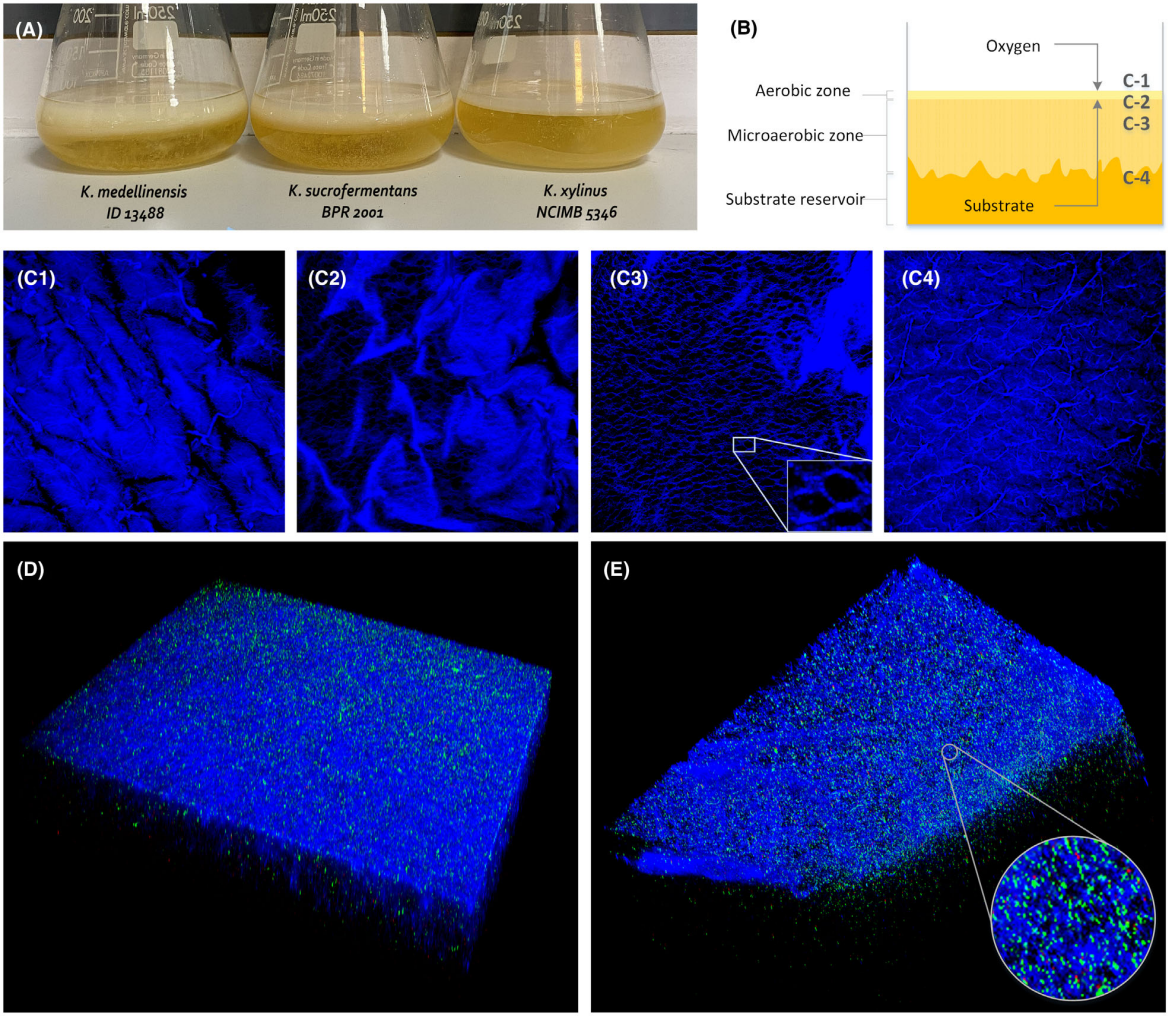
A. Bacterial cellulose being produced in static culture by three strains of the genus *Komagataeibacter* that yield different amounts of the biopolymer. B. 3D structure of BC in static culture. C‐1 to C‐4. Confocal laser scanning microscopy images acquired at the different depths of the BC membrane set in image B. D and E. Different views of a 3D reconstructed structure of a BC membrane being created by *K. medellinensis*. Images C–E were acquired through confocal laser scanning microscopy using Calcofluor White M2R to stain cellulose, which was coloured in blue and LIVE/DEAD™ BacLight™ kit having SYTO 9 and propidium iodide to stain living and dead cells, coloured in green and red, respectively.

Amongst the bacterial biopolymers, ELMs based on BC currently constitute the most prominent examples due to the exceptional properties of the BC natural hydrogels (Fig. 3). It has been suggested that the BC producers immobilize themselves in a 3D network to maintain a position of interface with the required nutritional intake, close to the air phase (Fig. 3). In the year 1988, Thompson *et al*. discovered tunnels presenting a regular orientation of a diameter of around 7 µm used by bacteria to move up and down (Fig. 3). Bacteria are thought to move to the aerobic zone in search of oxygen, and then start to produce cellulose; once the BC pellicle starts to impede the free disposition of substrate from bacteria, they move down to acquire nutrients whilst continuously forming cellulose fibres. Once they suffer from oxygen limitation, they move upwards again through the tunnels and restart the production cycle (see Fig. 3 *K. medellinensis* cells distributed with in the BC hydrogel). These tunnels, in combination with the high water retention capacity of the natural BC hydrogels, provide an ideal environment for the establishment of stable living cell systems. Another key advantage of using BC membranes as scaffolds for ELMs involves the facility for modification of their nanostructure and shape in order to create an adequate environment for the cells of interest and to increase their efficiency in the final application. Thus, BC can be synthesized in a variety of shapes through simple submersion of an object within the culture broth. Some examples, apart from the membrane shape obtained under static culture and the sphere beads under shaken culture, involve the use of microgroove moulds of agarose that produce grid‐shaped BC (Zang *et al*., 2014), the culture of bacteria with a tubular calcium alginate hydrogel that forms BC microstrands (Hirayama *et al*., 2013), or bacteria culture within a porous silicone tube, which forms tubular BC membranes (Putra *et al*., 2008). In order to provide better coating with BC of 3D objects with an irregular surface, a technique has been developed, which entails increasing the roughness and adherence of the item (Rühs *et al*., 2020). By adjusting culture time, they were able to replicate the piece as if they were moulds. The fact that BC‐producing bacteria move towards cavities of submerged objects and/or to their roughest areas has been attributed to the greater amount of oxygen that might remain available in these irregular surfaces, and to the tendency to immobilize of this type of bacteria. The porosity and pore size of BC membranes can also be adapted for use with the addition of compounds such as ferrofluid, hemicellulose, CS, potato starch or carboxymethylcellulose to the culture medium (Cheng *et al*., 2011), the application of a rotating magnetic field during BC synthesis or even by simple modification of the culture medium composition.

### Engineering BC‐producing bacteria and other living cargos for ELM production

Several approaches have been followed based upon genetically engineering BC‐producing bacteria to create a novel and functional class of BC‐based materials, which are of the greatest interest in the field of ELMs (Gilbert *et al*., 2021). Apart from varying the culturing conditions, as previously described, the chemical and physical properties of BC can be altered by genetically engineering the pathways of BC synthesis in BC‐producing strains. Florea *et al*. recently developed genetic toolkits for reprogramming *K. rhaeticus* iGEM for the production of cellulose‐based material. As a proof of concept, this toolkit enabled the production of intracellular proteins in *K. rhaeticus*, which became fluorescent in response to a small externally applied chemical inducer, which enabled cell detection for spatial patterning into the cellulose pellicle (Florea *et al*., 2016). The same toolkit has also been used to engineer bacteria with the use of an RNA‐based silencing system that represses UPGase, thus stopping BC production when a chemical inducer is externally applied. These genetic toolkits consist of standardized modular DNA sequences containing different origins of replication, inducible and constitutive promoters, fluorescent protein reporter genes, terminator sequences and ribosomal binging sites (RBSs) that can be used for genetic programming of bacteria.

Another example is the cell‐cell communication ability induced in *K. rhaeticus*, which allows the detection of nearby cells within the growing material and responds by triggering differential gene expression (Walker *et al*., 2019). The recently sequenced genomes of many BC‐producing strains in combination with the development of these new modular toolkits and the implementation of CRISPR and CRISPRi technologies offers many possibilities to genetically engineer BC‐producing bacteria and, therefore, to fabricate new ELMs fit for use in several fields (Huang *et al*., 2020).

A step forward in synthetic biology for the development of tools has enabled engineering microbial consortia to generate BC‐based ELMs by co‐culturing microorganisms with programmable functionalities. This new methodology has proved to have a great potential for developing ELMs with engineered and design capabilities. A promising synthetic biology strategy recently co‐cultured the engineered eukaryotic model yeast strain *Saccharomyces cerevisiae* and the BC‐producing bacteria *K. rhaeticus* (Gilbert *et al*., 2021). Genetically reprogrammed *S. cerevisiae* cells developed tailored functions in the BC‐living system such as secretion of proteins and sensing of chemical, physical and optical signals, consequently modifying the BC material properties (Gilbert *et al*., 2021).

Another interesting approach consists of co‐culturing *K. hansenii*, as a BC producer, and engineered *E. coli*, as a curli fibre producer, in droplets to produce hybrid living capsules. The encapsulated E. coli can produce engineered protein curli nanofibres within the BC matrix, yielding hybrid capsules capable of sequestering specific biomolecules from the environment and enzymatic catalysis. Furthermore, this ELM can alter its own bulk physical properties through enzyme‐induced biomineralization (Birnbaum *et al*., 2021).

A recent approach refers to BC‐based ELMs, which can detect damage and heal themselves (Caro‐Astorga *et al*., 2021). These ELMs have been fabricated in a modular fashion from millimetre‐scale biofilm spheroids grown by shaking cultures of engineered *K. rhaeticus*.

Finally, the application of prokaryotic phototrophs such as cyanobacteria is emerging as a promising alternative for sustainable development of ELMs. The autotrophic capabilities of cyanobacteria have tremendous potential for the development of cargos for ELMs as synthetic biology can be driven by CO_2_ and light. Several methods and new tools for genetically engineering cyanobacteria have been developed for a range of applications. For instance, advanced applications such as surface display systems in model cyanobacterial strains based on expression of genetically fused heterologous functional groups to outer membrane porin (SomA) (Fedeson and Ducat, 2017) has enabled cell‐cell adhesion systems with engineered *S. cerevisiae* or other functionalized molecules. Highly original biomineralization and successive regeneration of engineered living building materials has also been reported; this involves the use of photosynthetic cyanobacteria and an inert sand‐gelatine scaffold (Heveran *et al*., 2020). Another innovative example refers to the use of transgenic *Synechococcus* sp. PCC 7002 cyanobacteria (SynHA), which can produce oxygen and lymphangiogenic hyaluronic acid, in photosynthetic‐based biomaterials (Chávez *et al*., 2021).

Other examples include protoctista, such as *Chlamydomonas reinhardtii*, in the symbiotic consortium of ELMs for other aerobic bacteria to take advantage of the oxygen released by this type of organism, as in the case of BC‐producing bacteria. Definitively, BC might be employed for an enormous panel of smart applications including artificial leaves, photosynthetic bio‐garments, and adhesive labels (Balasubramanian *et al*., 2021).

## Conclusions

Bacteria are capable of naturally synthesizing a wide range of biopolymers such as polyamides, polysaccharides, polyesters and polyphosphates. Nonetheless, diversification thereof through metabolic engineering and chemical modification of their side chains or via cross‐linking with other biopolymers/molecules, provides even more potential functionalities. The present paper highlights the enormous non‐native diversity that can be generated from bacterial biopolymers through the synergistic combination of microbial biotechnology, synthetic biology, metabolic engineering, and materials science. Current trends point to the potential for bacterial biopolymers for use as scaffolds, which harbour living organisms, with the ultimate goal of creating ELMs, in which the material can self‐repair, and detect and respond to stimuli. However, not every bacterial biopolymer can meet the requirements for every living material. Alginate and PHAs showed constituted some of the first examples in the encapsulation of cells, and are, therefore, promising with regard to producing tailor‐made HLMs. But the adaptability of BC as a highly hydrated nanofibre network, along with its inherent features such as 3D structure control during the fermentation process, highlights BC‐based EMLs as the most promising system for the next generation of cutting‐edge materials. An important challenge facing researchers involves developing synthetic biology tools for engineering microorganisms as part of the EMLs, which have capacity to synthetize and secrete tailor‐made biomolecules (i.e. proteins, biopolymers or metabolites) in response to certain stimuli. Moreover, if the ELMs contain synthetic consortia, they will present unique features, which makes them an attractive platform for the creation of a broad range of cutting‐edge materials.

## Funding Information

Research on polymer biotechnology at the lab of Auxi Prieto is supported by the European Union’s Horizon 2020 Research and Innovation Programme under grant agreements no. 745737 (Afterlife), no. 760994 (Engicoin), no. 870294 (Mix‐Up), no. 814418 (SinFonia) and no. 101000733 (Promicon). At National level, the lab is supported by grants from the Community of Madrid (P2018/NMT4389), the Spanish Ministry of Science and Innovation under research grants BIOCIR (PID2020‐112766RB‐C21), REVOLUZION (PLEC2021‐008188) and the Juan de la Cierva aid of Cristina Campano (FJC2019‐040298‐I).
